# Features and Functions of the Conserved Herpesvirus Tegument Protein UL11 and Its Binding Partners

**DOI:** 10.3389/fmicb.2022.829754

**Published:** 2022-06-03

**Authors:** Linjiang Yang, Mingshu Wang, Anchun Cheng, Qiao Yang, Ying Wu, Juan Huang, Bin Tian, Renyong Jia, Mafeng Liu, Dekang Zhu, Shun Chen, Xinxin Zhao, Shaqiu Zhang, Xumin Ou, Sai Mao, Qun Gao, Di Sun

**Affiliations:** ^1^Research Center of Avian Diseases, College of Veterinary Medicine, Sichuan Agricultural University, Chengdu, China; ^2^Institute of Preventive Veterinary Medicine, Sichuan Agricultural University, Chengdu, China; ^3^Key Laboratory of Animal Disease and Human Health of Sichuan Province, Chengdu, China

**Keywords:** UL11, interaction, herpesvirus, viral cycle, secondary envelopment

## Abstract

The herpesvirus UL11 protein is encoded by the *UL11* gene and is a membrane-anchored protein with multiple functions. In the last stage of viral replication, UL11 participates in the secondary envelopment process. It also plays a key role in primary envelopment, the transportation of newly assembled viral particles through cytoplasmic vesicles, and virion egress from the cell. UL11 is an important accessory protein and sometimes cooperates with other proteins that participate in virus-induced cell fusion. Cell fusion is necessary for cell-to-cell transmissions. This review summarizes the latest literature and discusses the roles of UL11 in viral assembly, primary and secondary envelopment, and cell-to-cell transmission to obtain a better understanding of the UL11 protein in the life cycle of herpesviruses and to serve as a reference for studying other viruses. Additionally, some recently discovered characteristics of UL11 are summarized.

## Introduction

The *Herpesviridae* family is composed of *Alphaherpesvirinae*, *Betaherpesvirinae*, and *Gammaherpesvirinae*. The double-stranded DNA genome, capsid, tegument, and envelope form a mature herpesvirus virion (Guiping et al., 2007; Boštíková et al., 2014). The *Aphaherpesvirinae* subfamily includes many viruses, including human alphaherpesvirus-1/2 (HHV-1/2), also called herpes simplex virus-1/2 (HSV-1/2); pseudorabies virus (PRV); duck plague virus (DPV) (Qi et al., 2008; Zhao et al., 2008; Chang et al., 2009; Guo et al., 2009; Jia et al., 2009; Wu et al., 2012); human alphaherpesvirus-3 (HHV-3), also called varicella-zoster virus (VZV); equine herpesviruses (EHV); bovine herpesvirus (BoHV); infectious laryngotracheitis virus (ILTV); and Marek’s disease virus (MDV) (Kobty, 2015; Smith, 2017; You et al., 2017; Lefkowitz et al., 2018). The *Betaherpesvirinae* subfamily includes human alphaherpesvirus-5 (HCMV) and murine cytomegalovirus (MCMV). Human alphaherpesvirus-4 (HHV-4), also called Epstein–Barr virus (EBV), murine gammaherpesvirus-68 (MHV-68), and human alphaherpesvirus-8 (HHV-8) (Yuan et al., 2005), also called Kaposi’s sarcoma-associated herpesvirus (KSHV), are classified into the *Gammaherpesvirinae* subfamily (Foulon, 1992; Wu et al., 2020).

Tegument proteins are key components of herpesviruses (Newcomb et al., 2007). They are classified into two types, “inner” or “outer” tegument proteins, according to their preferential association with either the capsid or viral membrane during the viral lifecycle. When virions are lysed with non-ionic detergents, the inner or outer tegument proteins can be detected (Wolfstein et al., 2006; Mettenleiter et al., 2009; Radtke et al., 2010). Tegument proteins have multiple functions, including affecting viral replication by regulating gene transcription, destroying host innate immune responses, halting cell protein synthesis, and facilitating viral particle assembly by constructing a bridge that links viral capsids and envelope proteins (Nicoll et al., 2012; Owen et al., 2015), and evading innate immunity, as previously summarized (Yang et al., 2019).

Herpesviruses display two characteristic replication modes, namely, a rapid, productive replication cycle and a life-long quiescent infection (Zeev-Ben-Mordehai et al., 2014; You et al., 2017). To ensure lytic replication, after a virus has entered a cell, capsid DNA is released into the nucleus, and then viral DNA begins to replicate. After assembly and genome packaging, the intact capsid leaves the nucleus, and it immediately undergoes primary and de-envelopment at the nuclear envelope, followed by tegumentation and secondary envelopment in the cytoplasm. Ultimately, mature virions are released from infected cells by exocytosis (Yang et al., 2019).

The UL11 protein and its homologs are essential to the viral replication cycle, especially for cell-to-cell transmissions and, particularly, the secondary envelopment process (Yang et al., 2020a). In recent years, UL11 functions have been characterized (Kopp et al., 2004; Koshizuka et al., 2006; Yang et al., 2021, 2022). However, the roles played by herpesvirus UL11 in the viral replication cycle have not been fully elucidated. This article reviews the characteristics and roles of herpesvirus UL11 in the viral life cycle, especially its cell-to-cell transmission and cell fusion functions and UL11 interaction partners. On the basis of UL11 studies, we propose hypotheses to test and describe problems that remain to be solved to further characterize the importance of UL11 in future studies.

## The UL11 Protein

Approximately 700 copies of UL11 proteins per HSV-1 particle have been detected (Loomis et al., 2006). Using circular dichroism, limited proteolysis, small-angle X-ray scattering (SAXS), liquid–liquid phase separation (LLPS) analysis, and light microscopy, researchers identified that UL11 *in vitro* has an intrinsically disordered (ID) structure (Metrick et al., 2020). UL11 and its homologs in virions have been classified as tegument components (Johannsen et al., 2004; Tomtishen, 2012). The molecular size and localization of UL11 differ in herpesvirus species (Loomis et al., 2006). For example, the molecular size of UL11 is 15 kDa in HSV-1 (McGeoch et al., 1988), the molecular size of the UL11 homolog ORF51 in EHV-1 is 12 kDa (Badr et al., 2018), and the molecular size of UL11 in DPV (Yang et al., 2021) is approximately 15 kDa. In VZV, the ORF49 encoded protein is 13 kDa (Sadaoka et al., 2007). In β-herpesviruses, the molecular sizes of HCMV UL99 and MCMV UL99, each encoding pp28 protein, are 28 and 11.8 kDa, respectively (Cranmer et al., 1994; Hitomi et al., 1997). In γ-herpesviruses, the ORF38 protein in KSHV has been detected as 10 kDa (Wu et al., 2016). A 12-kDa protein has been found in MHV-68-infected cell lysate, which is the molecular size of ORF38 (Efstathiou et al., 1990). Interestingly, in all herpesviruses, UL11 expression partially overlaps with UL12 expression (Adam et al., 1995; Johannsen et al., 2004). Although HCMV pp28 has been shown to multimerize without other viral proteins, the researchers of this study did not rule out the possibility of an interaction with a cellular protein following pp28 multimerization (Seo and Britt, 2008). Table 1 shows detailed information about the sizes of the UL11 protein in various herpesviruses.

**TABLE 1:** Features of herpesvirus UL11 gene and homologs.

Subfamily	Virus name	Gene	Coding protein	Number of amino acids	Gene type
*Alphaherpesvirinae*	HSV-1	UL11	UL11	96	L
	HSV-2	UL11	UL11	96	L
	VZV	ORF49	ORF49	81	L
	EHV-1	ORF51	ORF51	74	E/L
	DPV	UL11	UL11	87	L
	BoHV	UL11	UL11	65	L
	PRV	UL11	UL11	63	ND
	MDV-2	UL11	UL11	81	ND
	ILTV	UL11	UL11	80	L
*Betaherpesvirinae*	HCMV	UL99	pp28	190	E/L or L
	MCHV	UL99	pp28	112	L
*Gammaherpesvirinae*	MHV-68	ORF38	ORF38	75	IE
	EBV	BBLF1	BBLF1	75	ND
	KSHV	ORF38	ORF38	61	ND

### Post-translational Modification of the UL11 Protein

The UL11 protein undergoes multiple forms of post-translational modification, namely, myristoylation, and palmitoylation. Covalent modification with a fatty acid is an established characteristic in several cell types and viral polypeptides (Sefton and Buss, 1987), and palmitoylation and myristoylation are two fatty acid modifications. Studies on α-herpesviruses have shown that HSV UL11 and VZV ORF49 are myristoylated with myristic acid (MacLean et al., 1989; Harper and Kangro, 1990; Sadaoka et al., 2007). Additionally, EHV-1 UL11 has a conserved myristylation consensus sequence (M-G-X-X-X-S/T) (Resh, 1999). In β-herpesviruses, HCMV UL99 encodes a tegument protein that undergoes myristoylation (Chee et al., 1990). In KSHV, ORF38 is also myristoylated (Sanchez et al., 2000). In addition to myristoylation, HSV-1 UL11 is palmitoylated. The palmitoylated modification sites in UL11 contain one or more of three consecutive cysteines in the UL11 amino terminus (N-terminus) (Loomis et al., 2001). UL11 homologs of HSV-1, HCMV, EBV, and likely HSV-2 are also palmitoylated (Baird et al., 2008; Seo and Britt, 2008; Chiu et al., 2012; Jones and Lee, 2004).

In addition, UL11 undergoes phosphorylation. The HSV-1 UL11 homolog—HCMV UL99 phosphoprotein—has been identified strictly as a late kinetic reaction product (Mocarski and Courcelle, 2001). Studies have suggested that HCMV pp28 phosphorylation contributes to its intracellular trafficking and efficient viral assembly and incorporation. For example, when protein phosphatase 2 (PP2A) modified pp28, the mobility of pp28 was altered in SDS–PAGE, indicating that pp28 was indeed phosphorylated, potentially on several residues (Seo et al., 2020). Moreover, phosphorylation of pp28 tyrosine 34 and serines 41–43 was found to be important for transfected pp28 trafficking to the endoplasmic reticulum-Golgi-intermediate compartment (ERGIC) (Seo and Britt, 2006; Seo et al., 2020). Another study reported that deletion of HCMV UL26 caused pp28 hypophosphorylation, suggesting that the UL26 protein affected the normal phosphorylation of pp28 in virions and possibly additional tegument proteins (Munger et al., 2006). In MCMV, pp28 has been found to be phosphorylated, potentially by protein kinase C and casein kinase II (Cranmer et al., 1994), with amino acids (aa) 25–28 and 91–94 of pp28 being the protein kinase C targets and aa 42-47 being the casein kinase II targets (Cranmer et al., 1994). For HCMV, pp28 is the autophagy-initiating protein kinase ULK1 phosphorylation target (König et al., 2021). Table 2 contains information on these post-translational modifications.

**TABLE 2:** UL11 and homolog post-translationally modified sites.

Virus	Protein	Post-translational modification and enzyme	Modification sites
**Myristoylation modification**			
HSV	UL11	By myristic acid	N-terminus M-G-X-X-X-S/T consensus sequence
VZV	ORF49		
EHV-1	UL11		
KSHV	ORF38		
HCMV	pp28		
**Palmitoylation modification**			
HSV	UL11	By palmitic acid	One or more of three consecutive cysteines in UL11 N-terminal
HCMV	pp28		Multiple cysteine residues
EBV	BBLF1		Cys-8
**Phosphorylation modification**			
MCMV	pp28	By potential protein kinase C	aa 25–28 and 91–94
		By casein kinase II	aa 42–47
HCMV	pp28	By ULK1	Not determined
		By UL26	Not determined
		By PP2A	aa 34 and 41–43

### Localization of the UL11 Protein

The localization of the UL11 protein is dynamic. In HSV-1, UL11 has been shown to be located at membranes, including nuclear and Golgi-derived membranes, and especially at the trans-Golgi network (TGN) (Baines et al., 1995; Loomis et al., 2001). In EHV-1, UL11 was predominantly localized at the TGN in infected cells, while in transfected cells, UL11 was localized at the plasma membrane, as determined through confocal laser scanning microscopy (Schimmer and Neubauer, 2003). Similar to UL11 in EHV-1, the virion component VZV ORF49 localized predominantly at the TGN in infected cells (Sadaoka et al., 2007). In DPV, UL11 is localized at the perinuclear area in infected cells and membranes, especially at the TGN membranes, in transfected cells (Yang et al., 2021). HCMV pp28 is localized in the ERGIC, which is a dynamic compartment in the secretory pathway that interfaces with both the ER and the Golgi apparatus (Sanchez et al., 2000). Another study identified MHV-68 ORF38 as a tegument protein that localized to cytoplasmic compartments (CT) during transient transfection and viral infection (Shen et al., 2014). Furthermore, EBV BBLF1 is localized to the TGN and perinuclear areas (Chiu et al., 2012). Hence, in most herpesviruses, UL11 is localized in the Golgi apparatus.

### Gene Type of UL11

When a target cell is infected by herpesviruses, viral genomic DNA is circularized, and a temporal cascade of viral gene transcription is initiated; this cascade consists of three stages: immediate-early (IE), also called the α gene; early (E), also called the β gene; and late (L), also called the γ gene, indicating the expression period and dependence on other gene products (Mahmoudian et al., 2012; Yang et al., 2020b). ILTV UL11 has been identified as an L gene in alphaherpesviruses. Similarly, UL11 is also an L gene in HSV-1 and BoHV (Baines and Roizman, 1992; Desloges and Simard, 2001). In DPV and VZV, UL11 is also an L gene, as determined by its late-stage expression (Sadaoka et al., 2007; Yang et al., 2021). In EHV-1, UL11 is an early-late gene (Schimmer and Neubauer, 2003). Researchers characterized the HCMV pp28 upstream (pp28US) promoter with two regulatory components, one dependent on the onset of viral DNA synthesis and the other replication-independent, responding to viral trans-acting factors (Wu et al., 2001). Kohler et al. (1994) found that the pp28US promoter was sufficient to establish UL99 late kinetics (Kerry et al., 1997). In contrast, Jones and Lee showed that UL99 was expressed as an early-late protein, not an L protein, similar to UL99 in EHV-1 (Jones and Lee, 2004). MCMV UL99 has also been classified as an L gene based on its gene expression kinetics (Cranmer et al., 1994). Homologs of HSV-1 UL11 gene products in the herpesvirus family are nearly all conserved tegument proteins (Baer et al., 1984; Davison and Scott, 1986; Chee et al., 1990; Telford et al., 1992; Dijkstra et al., 1997; Osterrieder, 1999; Shen et al., 2014). Surprisingly, in contrast to other herpesvirus genes, MHV-68 ORF38 has been designated an IE gene after treatment with cycloheximide (Ebrahimi et al., 2003). Detailed information can be found in Table 1.

## Functions of UL11 in the Viral Life Cycle

### Viral Replication of the UL11 Null Mutant

The effect of the UL11 deletion on viral replication differs by virus species. In HSV-1 and PRV, the absence of UL11 resulted in a 10-fold reduction in viral titer (Baines et al., 1995; Kopp et al., 2003; Fulmer et al., 2007), and neither myristoylation nor palmitoylation modification of HSV-1 UL11 was found to be necessary for viral replication (MacLean et al., 1992; Baird et al., 2010). In EHV-1, deletion of UL11 in the neuropathogenic strain Ab4p resulted in abrogated progeny production. That is, in these mutant viruses, UL11 is replicated only in complementing cells; hence, UL11 is considered to be essential for EHV-1 (Badr et al., 2018). In addition, the absence of UL11 in the EHV-1 RacH and RacL22 strains, which exhibited one-step growth kinetics and were used to infect RK13 cells, resulted in an approximate 10- to 20-fold reduction in intracellular and extracellular virus titers (Schimmer and Neubauer, 2003). The reasons for the differences in these results were discussed in the article (Badr et al., 2018): First, the Ab4p UL11 sequence is the same as that in the RacH strain (data not shown); however, the restriction patterns of UL11 expression in the RacH and RacL22 strains differ from that in the Ab4 strain, which has a shorter left UL terminus and lacks a *Bam*HI site (Hubert et al., 1996). Second, 0.85 kbp sequences in the inverted repeat regions are deleted in both copies of RacH (Hubert et al., 1996). Moreover, other viral proteins may compensate for the reduction in UL11 function in the Rac strain but not in Ab4p. Additionally, there may be experimental shortcomings in one of the studies. Similarly, HCMV UL99 is essential for the production of infectious viruses (Silva et al., 2003; Seo and Britt, 2007). No infectious progeny viruses were detected in fibroblasts infected with an HCMV pp28-deficient mutant, which also showed impaired viral replication due to the lack of enveloped virus particles. Additionally, although ORF49 is not essential for viral replication (Sadaoka et al., 2007), the VZV titer of the cell-free virus was 3 to 5% of that of the control virus (Sadaoka et al., 2014). In contrast to the effect of deletion of the essential gene UL99 in HCMV, deletion of BBLF1 only partially impaired EBV lytic replication (Chiu et al., 2012), reducing the production of EBV particles by approximately 54%. In another γ-herpesvirus, KSHV, ORF38 has not been considered essential for virus replication; however, the viral yield of the ORF38-null mutant was reduced 10-fold (Wu et al., 2016).

### UL11 Influences Primary and Secondary Viral Envelopment

When the capsid is intact, it undergoes primary envelopment. The specific process of primary envelopment involves intranuclear capsid budding at the inner nuclear leaflet, and later, fusion with the outer leaflet of the nuclear membrane. Specifically, after primary envelopment, capsids undergo nuclear membrane fusion and de-envelopment, and then the nucleocapsids bud from the nucleus and are transported to the TGN, where the tegument is formed and where the nucleocapsid undergoes secondary envelopment (Mettenleiter, 2002).

For HSV-1, the incidence of UL11-deleted capsid juxtaposition with the inner lamellae of nuclear membranes has been shown to be greater than that of wild-type or repaired viruses. Specifically, approximately 3% of the virion particles in the nuclei of cells infected with wild-type or repaired viruses were in contact with the inner lamellae, whereas in cells infected with the mutant virus, 11% of the nuclear capsids were in contact with the inner lamellae. Moreover, the number of capsids partially enveloped by the inner lamellae of cells infected with the UL11-deleted virus was increased. In cells infected with wild-type, UL11-null or repaired viruses, enveloped particles, and, to a lesser extent, unenveloped particles accumulated in the space between the lamellae of the nuclear membrane 24 h after infection. The numbers of particles accumulating in this space were similar to the number of cells infected with each virus. This study suggested that UL11 gene deletion affected envelopment at the nuclear membrane by either slowing or inhibiting the process but had no effect on viral transit into the perinuclear space (Baines and Roizman, 1992). During the secondary envelopment stage, the HSV-1 UL11-null mutant produced fewer enveloped virions and more unenveloped CT capsids (Fulmer et al., 2007). In addition, deletion of PRV UL11 resulted in unenveloped capsid accumulation in the cytoplasm, which was inconsistently associated with recruited tegument proteins, similar to the effect of gE/gI and gM deletion mutants. Moreover, the absence of UL11 profoundly impaired the architecture of the Golgi-derived membrane, which is the typical site of secondary envelopment (Kopp et al., 2004). Similarly, when RK13 cells were infected with PRV UL11 and the UL51 double-deletion mutant, the morphology of the intracytoplasmic membranes was distorted, and nucleocapsids accumulated in the cytoplasm in association with aggregated tegument (Klupp et al., 2005b). The VZV ORF49-deletion mutant and the wild-type virus exerted the same effects (Sadaoka et al., 2007) in MRC-5 cells, as determined by ultrastructural analysis. This result suggests that VZV ORF49 did not affect primary or secondary envelopment in MRC-5 cells. In HCMV pp28-null mutant-infected cells, normal levels of viral DNA and L proteins were observed, but a large number of tegument-associated capsids accumulated in the cytoplasm and failed to undergo envelopment (Silva et al., 2003; Britt et al., 2004). Moreover, EBV BBLF1 has been hypothesized to be important for tegumented capsid budding into glycoprotein-embedded membranes during viral maturation (Chiu et al., 2012). MHV-68 ORF38 has also been reported to participate in secondary envelopment (Shen et al., 2014). As shown in a membrane flotation assay, KSHV ORF38 participated in the virion packaging process, promoting virion packaging into cytoplasmic vesicles critical for viral maturation and egress (Wu et al., 2016). Detailed information is presented in Table 3.

**TABLE 3:** Comparison of viral UL11 necessity for different viruses and morphologies.

Different UL11-null mutants	Virus name	Whether an essential gene	Morphology of these UL11-deleted mutants
*Alphaherpesvirinae*	HSV-1	No	Fewer enveloped virions and two- to three-fold increase in unenveloped cytoplasmic capsids
	PRV	No	Unenveloped capsids accumulated in the cytoplasm, tegument proteins aggregated, and distortion of the architecture of Golgi-derived membranes
	EHV-1	?	Remained unclear
	VZV	No	No difference between the wild-type and the UL11 deletion mutant
*Betaherpesvirinae*	HCMV	Yes	Tegument-associated capsids accumulated in the cytoplasm that failed to acquire an envelope
*Gammaherpesvirinae*	MHV-68	ND	ND
	EBV	No	Tegumented capsids accumulated
	KSHV	No	Viral DNA present in the vesicle-rich fractions was reduced

### Effect of UL11 on Trafficking

Similar to that in another herpesvirus model, in an HSV-1 model, assembled nucleocapsid shuttling into the nucleus involved separate inner and outer nuclear membrane budding-fusion events; subsequently, these nucleocapsids moved through the cytoplasm as unenveloped capsids and underwent envelopment at TGN-derived vesicular membranes. As the nucleocapsids acquired this final lipid bilayer at the TGN, the tegument, which includes glycoproteins, was formed (Browne et al., 1996; Loomis et al., 2001). Notably, many other molecules are involved in the process of transporting non-enveloped viral particles such as microtubules.

The detected correlation between UL21 and microtubules (Takakuwa et al., 2001) has raised the possibility that UL21 participates in capsid transport to TGN-derived vesicles (Guo et al., 2010), where UL16 interacts with UL11, facilitating the budding process by linking capsids to the membrane (Loomis et al., 2003). A study showed that palmitoylation of HSV-1 UL11 was necessary for both Golgi targeting specificity and effective membrane binding. In addition, one-half of a conserved acidic sequence cluster in UL11 has been found to be important for tegument protein recycling from the plasma membrane to the Golgi apparatus (Loomis et al., 2001). HCMV UL99 has been shown to traffic to vacuole-like cytoplasmic structures, and the second glycine and a 44–57 acid sequence have been suggested to be necessary for envelopment (Jones and Lee, 2004). Further analyses of ORF33/ORF38 mutants revealed that the production of virion-containing vesicles was decreased, indicating that KSHV ORF38 facilitated the transport of newly assembled viral particles in cytoplasmic vesicles, a process that is essential for viral maturation and egress (Wu et al., 2016). When the first 100 amino acids in respiratory syncytial virus (RSV), Gag proteins were replaced by the entire UL11 sequence; the resulting chimeric protein was targeted in the Golgi apparatus rather than being localized to the plasma membrane. Studies have shown that a region consisting of the first 49 amino acids in UL11 is myristoylated, which is required for Golgi-specific targeting. In addition to the identification of a previously unknown modification site on UL11, these experiments showed that UL11 directed RSV Gag to internal cellular membranes (Bowzard et al., 2000). Taken together, these studies suggest that UL11 has highly dynamic membrane-trafficking properties.

### Effect of UL11 on Cell Fusion and Cell-to-Cell Spread

Viruses spread to uninfected cells in two ways (Carmichael et al., 2018). Upon release from an infected cell and entry into the surrounding environment, a virus enters a new cell through cell-free spread. This modality describes both the viral infection of new hosts and transmission between cells within a host. However, some virions, including those of all herpesviruses, pass directly through cell junctions, enabling virion detection by neutralizing antibodies (Johnson and Huber, 2002; Mateo et al., 2015; Carmichael et al., 2018). In addition, virus-induced cell fusion, which facilitates transmission into adjacent cells, also protects virions from being exposed to neutralizing antibodies. This model of infectivity is observed in herpetic lesions (Roizman et al., 2007). This modality of transmission is important and worth studying because the mechanisms of cell-to-cell spread and cell fusion in herpesvirus infections remain unclear.

The mechanism of cell-to-cell spread is complex. In HSV-1, gE is crucial for cell-to-cell spread, as indicated by the inhibition of cell-to-cell spread in gE-deletion mutants treated with neutralizing antibodies *in vitro* and transmission between neurons in mouse models (Dingwell et al., 1994; Howard et al., 2014). Further research showed that the peripheral membrane-binding protein UL11, which is associated with tegument proteins UL16 and UL21, forms a complex on the cytoplasmic tail of gE. In cell culture, this complex appears to be critical for cell-to-cell spread (Han et al., 2012; Sarfo et al., 2017). The replication efficiency of the HSV-1 UL11-null mutant was nearly one logarithmic unit less than that of the wild-type virus, and the UL11-null virus formed plaques that were, on average, one-third the size of those created by the wild-type virus (Kim et al., 2014). Other HSV-1 proteins, including UL51, gI, and UL34, have also been shown to participate in cell-to-cell spread (Dingwell and Johnson, 1998; Haugo et al., 2011; Roller et al., 2014). When UL11 was deleted in EHV-1, the viral plaque decreased significantly—to approximately 20% of the parental virus (Schimmer and Neubauer, 2003). Moreover, the VZV UL11-null mutant showed diminished plaque compared to the wild-type strain, similar to its effect on HSV-1 infection of the human malignant melanoma cell line. However, no differences in plaque size or cell-to-cell spread were observed in MRC-5 human embryonic fibroblasts infected with the wild-type or mutant virus (Sadaoka et al., 2007).

Surprisingly, UL11 was also shown to be vital for the virus-induced cell fusion process. In the alphaherpesvirus HSV-1, when UL11 and gM were deleted, the virus entered cells more slowly than the wild-type virus, indicating that gM and UL11 both regulate the HSV-1 membrane fusion machinery during virus-induced cell fusion and virus entry (Kim et al., 2013). Cell fusion was induced when a 28 aa sequence in the carboxyl-terminus (C-terminus) of gB was deleted by the insertion of a stop codon and when the gK alanine residue at position 40 was replaced with valine. Among gB syncytial mutants, the gBΔ28 mutation is considered to exert the most profound inductive effect on the fusion of most cell types in tissue culture (Silverman et al., 2012). In addition, the gKsyn20 mutation caused extensive virus-induced cell fusion of most cell types (Chouljenko et al., 2009). Abrogation of UL11 led to severe impairment of gB△28 and gKsyn20-induced virus-induced cell fusion. In addition, UL11 affected gBA855V-induced cell fusion, confirming the aforementioned result that suggests that UL11 is required for gB△28 induced cell fusion (Silverman et al., 2012; Kim et al., 2013). In a study by Han, the UL11-null mutant in the presence of the UL11 interaction proteins UL16 and UL21 failed to form syncytia in HSV-1 (Han et al., 2012).

### Effect of UL11 on Virion Egress

**FIGURE 1:**
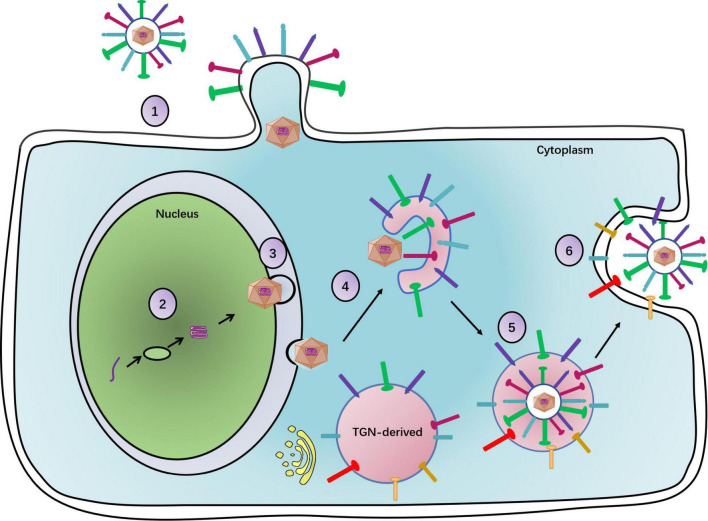
The complete process of the herpesvirus life cycle. (1) Viral particles enter a cell. With gM, UL11 promotes virus entry. (2) The viral DNA replication. (3) Primary envelopment of viral particles. UL11 participates in capsid primary envelopment. (4) Primary envelopment capsids arrive at the TGN to obtain secondary envelopment. UL11 can raise glycoproteins at the TGN to carry on secondary envelopment. (5) Secondary enveloped viral particles will be transported by the vesicle. UL11 promotes the association of the newly assembled viral particles with the vesicle. (6) Mature viral particles with the vesicle egress out of a cell. UL11 increases the number of viral particles released from the cell.

Upon maturation, viral particles are released from host cells. UL11 plays a key role in viral egress, as indicated by the profound delay in UL11-null mutant HSV-1 virus release from infected cells. Specifically, the wild-type virus showed an approximately 500-fold increase in virion release, while the UL11-null mutant showed only a 15-fold increase between 8 and 14 h post-infection (Baines and Roizman, 1992). Moreover, in the HSV-1 UL11 null mutant, Chouljenko et al. (2012) found that there were fewer extracellular virions than cytoplasmic virions. Interestingly, HCMV pp28 was phosphorylated by the autophagy-initiating protein kinase ULK1, which leads to efficient viral release (König et al., 2021). Thus, UL11 may facilitate the viral egress process. Figure 1 depicts a function map for the UL11 protein.

## UL11 Interaction Partners

### The gE-UL11-UL16-UL21 Complex

The classical interaction between UL11 and UL16 has been determined to be conserved in all members of the *Herpesviridae* family (Fossum et al., 2009; Diefenbach, 2015). Evidence has shown that the interaction between HSV-1 UL11 and UL16 may promote the correlation observed between nucleocapsids and the secondary envelopment process (Vittone et al., 2005; Meckes and Wills, 2007; Meckes et al., 2010; Johnson and Baines, 2011; Starkey et al., 2014). Because it is a nucleocapsid-associated protein, upon interaction with its partners, UL16 may recruit nucleocapsids to cytoplasmic membranes for envelopment within infected cells. In PRV, the interaction between UL11 and UL16 was identified in a glutathione S-transferase (GST) pull-down assay (Harper et al., 2010), and the complex promoted UL36 incorporation into mature virions (Michael et al., 2006), which was also observed in VZV (Sadaoka et al., 2014). Furthermore, binding with HCMV UL99 stabilized the UL16 homolog UL94 (Phillips et al., 2012), facilitating cytoplasmic nucleocapsid transport to membranes (Phillips and Bresnahan, 2012). In KSHV, ORF33 interacts with ORF38, and this interaction leads to the optimal production of infectious progeny viruses (Wu et al., 2016). The UL16-binding sites in homologs of HSV-1 and HCMV have been confirmed to be in the N-terminal half of the UL11 protein (Yeh et al., 2008; Liu et al., 2009): specifically, in HSV-1, they are in the acidic cluster sequence and leucine-isoleucine (LI) motifs within the UL11 N-terminus (Yeh et al., 2008). Similarly, in EBV, the interaction between BGLF2 and BBLF1 is required for the efficient production of infectious virus particles (Hung et al., 2020). These findings show that the interaction between the UL16 and UL11 proteins is conserved in the herpesvirus family.

**FIGURE 2:**
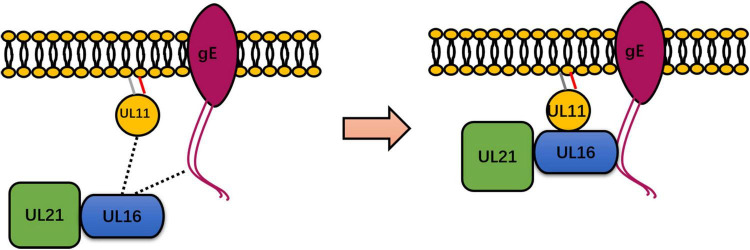
Summary of the known interactions between UL11 and UL16, UL21, and gE. UL11 binds to UL16, which is directly conserved in a representative member of *Herpesviridae*. However, UL11 formed a strong interaction with gE only in a study of HSV-1 and DPV. In HSV, DPV, and PRV, UL21 interacts directly with UL16. The dotted line represents the weak interaction between UL11 and UL16 and between UL16 and gE (left). UL16 may undergo enhanced binding with gE in the presence of UL11, UL16 may undergo enhanced binding with UL11, and UL16 may undergo enhanced binding with UL11 in the presence of UL21 in HSV-1 (right). Therefore, UL11, UL16, UL21, and gE form a complex. Some inspiration for this figure was obtained from previous articles (Han et al., 2012).

Although both gE and pUL21 are unique to the *Alphaherpesvirinae* subfamily and have not yet been investigated, homologs of pUL11 and pUL16 may bind to the cytoplasmic tail of envelope proteins in the Beta and *Gammaherpesvirinae* subfamilies. Notably, a study reported that an HSV-1 UL11 acidic cluster motif is indispensable for gE packaging (Han et al., 2011). In DPV, research has shown that DPV gE and UL11 preferentially and exclusively interact. UL11 can interact with the CT and ET domains of gE, and the CT domain of gE is critical for UL11 incorporation into a viral particle (Yang et al., 2021). In HSV-1, UL11, and UL16 have been shown to interact with the cytoplasmic tail of gE, and UL11 has been shown to initiate the weak interaction observed between UL16 and gE (Farnsworth et al., 2007; Han et al., 2011, 2012; Yeh et al., 2011). In HSV, DPV, and PRV, UL21 can interact with UL16 (Klupp et al., 2005a; Harper et al., 2010; Gao et al., 2017; Yang et al., 2020b). In HSV, UL21 interacts with UL11 in the presence of UL16, and UL21 promotes the weak interaction between UL11 and UL16 (Han et al., 2012). Similar to the UL11-gE interaction, the UL11 and UL21 interaction has been found to be weak and has been identified only in the *Alphaherpesvirinae* subfamily (Diefenbach, 2015). UL21 and UL16 are capsid-associated proteins, and UL11-UL16-UL21 can form a complex. Therefore, UL11 may indirectly affect capsid function. In fact, UL11, UL16, UL21, and gE may have formed a quadruple complex in transfected Vero cells (Han et al., 2012). Table 4 contains additional information, and details of the relationship between UL11, UL16, UL21, and gE are presented in Figure 2.

**TABLE 4:** Interaction domain between UL11 and other proteins.

Interaction	Subfamily	*Herpesviridae* member	Interaction domain
UL11-UL16	*Alphaherpesvirinae*	HSV-1	Leucine-isoleucine and acidic cluster motifs of UL11
		VZV	Phenylalanine 129, and four amino acids in the carboxyl-terminal half of the acidic cluster in ORF49
	*Betaherpesvirinae*	HCMV	Amino acids 22-43 of pp28
		MCMV	The non-conserved N-terminal region of UL94 has also been shown to contribute to the binding of UL99
	*Gammaherpesvirinae*	EBV	N-terminal region of BGLF2 is important to its interaction with BBLF1
		KSHV	Cysteines of ORF33 are involved in its interactions with ORF38
		MHV-68	Not determined
UL11-gE	*Alphaherpesvirinae*	HSV-1	C-terminal 26 residues of UL11
		DPV	Not determined the interaction domain
UL11-UL16-UL21	*Alphaherpesvirinae*	HSV-1	UL21can interact with UL11 when the UL16 exists; only determined in HSV-1
U11-UL56	*Alphaherpesvirinae*	HSV-2	Interaction only determined in HSV-2
UL11-gD	*Alphaherpesvirinae*	HSV-1	Interaction only determined in HSV-1
UL11-UL11	*Betaherpesvirinae*	HCMV	43aa; only determined in HCMV

### UL11 Can Form Homomultimers or Interact With Other Viral Proteins

Through GST pull-down assay and other immunoprecipitation experiments, Koshizuka et al. confirmed that UL11 interacted with UL56 in HSV-2-infected cells. UL56 is a tail-anchored type II membrane protein associated with the Golgi apparatus and cytoplasmic vesicles (Koshizuka et al., 2006). However, no reports have indicated that UL11 interacts with UL56 in other herpesviruses. On the basis of an immunoprecipitation assay, another study revealed that the CT domain of HSV-1 gD is bound to UL11 in HSV-1-infected cells (Farnsworth et al., 2007), but whether this interaction was direct or not was unclear, and it could not be induced in cells infected with other herpesviruses.

**FIGURE 3:**
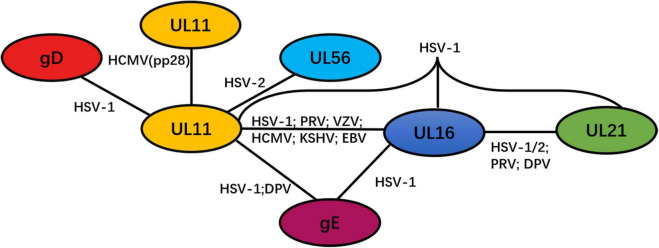
Network of protein–protein interactions between the tegument protein UL11 and other viral proteins. Black solid lines represent interactions.

UL11 can undergo self-interaction to form homomultimers. A study found that the initial 43 amino acid sequences in HCMV pp28 contain a domain that is critical for the self-interaction of pp28 (Seo and Britt, 2008). Although this interaction was found to be independent of other viral proteins, the possibility of a pp28 interaction with cellular proteins was not ruled out. Additionally, multimerization of UL11 has only been reported in HCMV. Detailed information on the UL11 network is presented in Figure 3.

### UL11 Can Interact With Lipid Rafts

Lipid rafts, also called detergent-resistant membranes (DRMs), are microdomain structures that are rich in cholesterol and glycosphingolipids. Although lipid rafts are generally located on the plasma membrane, rafts are often assembled in the Golgi apparatus (Simons and Ikonen, 1997). Many proteins interacting with lipid rafts have important roles in trafficking, signal transduction, and pathogen entry and exit (Koshizuka et al., 2007). Researchers found that HSV-2 UL11 was associated with lipid rafts. In DPV research, UL11 has been shown to interact with lipid rafts, and this interaction depends on the second glycine of UL11 (Yang et al., 2022). The dual acylation of UL11 is required for lipid raft association because mutated myristoylation or palmitoylation sites block lipid rafts (Koshizuka et al., 2007; Baird et al., 2008). Through an immunoprecipitation assay, a study elucidated the DRMs associated with HSV-1 UL11 and showed that they depended on LI and acidic cluster motifs in UL11 (Baird et al., 2008).

## Other Functions of UL11

Studying HSV-1, researchers found that the C-terminus of UL11 has a disordered structure, and further experiments indicated that this disordered structure fails to bind ribosomal RNA (rRNA) (Metrick et al., 2020). Additionally, this disordered structure was found not only in UL11 but also in all the tegument proteins of HSV-1. Moreover, this was the first report showing that UL11 is associated with RNA. Hence, the disordered tegument structure and its correlation with assembly mechanisms should be taken into account. The structural disorder in other tegument proteins needs to be further investigated because the details of these protein interactions are unclear.

For the study of HCMV, the UL99 transcript has been identified as a rapid marker of therapy effectiveness. Through a qRT–PCR assay analysis of 18 bronchoalveolar lavage samples from patients who were or were not subjected to antiviral treatment *in vivo* and *vitro*, UL99 has been developed as an antiviral control (Gambarino et al., 2014). In the past, it was difficult to study L gene promoters, as they were expressed in a disorderly fashion in transient assays (Kohler et al., 1994). However, a study of the entire viral genome showed that sequences from position –40 to +106 of the pp28 promoter are important for the L gene expression. The –6 to +46 sequences of the pp28 promoter repressed gene expression. The +46 to +88 sequence of the pp28 promoter was not particularly important to gene expression compared with the influences of the other sequences. These results indicate that translation of the L genes can be regulated by leader sequences in the pp28 gene (Kerry et al., 1997).

## Conclusion

Among all α*-*, β*-*, and γ*-*herpesviruses, UL11 is a conserved gene. In the *Herpesviridae* family, the viral protein–protein interaction network is dynamic and necessary for the secondary envelopment process. Since it can associate with multiple proteins, UL11 is a key mediator in this interaction network. Moreover, the UL11 interaction network is important for the secondary envelopment process. Future studies on the structure of the conserved interactions, such as the UL11-UL16 interaction, will facilitate further research in herpesvirus assembly and tegument structure. The mechanism and function of the UL11, UL16, UL21, and gE quadruple complex also need to be investigated. Details on the function of the UL11 interaction with the two types of capsid-associated proteins need to be ascertained to determine whether they are related to the construction of a bridge between the capsid and the envelope. Studies of the interaction between the host protein and UL11 are rare, and elucidating the UL11-host cell protein interactions within virions may reveal the question regarding whether host cell proteins are simply passively acquired during viral assembly and trafficking or whether other mechanisms are involved is worth investigating. For research on β*-*herpesviruses, UL99 has been determined to be an important factor in antiviral therapy research, and it may be useful as an additional tool for overcoming drug resistance at the onset of infection.

In addition to the UL11 interaction partners that regulate the viral life cycle, other functions need to be investigated. Most tegument proteins, such as UL41, UL48, and UL49, have been reported to participate in innate immunity. However, because research into the effect of UL11 on innate immunity is rare, it remains unclear whether or to what extent UL11 is needed or functional. Investigating the role played by UL11 in immunity will lay a foundation for UL11 function research.

## Author Contributions

LY conceived, designed, and drafted the manuscript. MW conceived and supervised the review. AC, QY, YW, JH, BT, RJ, ML, DZ, SC, XZ, SZ, XO, SM, QG, and DS approved the final manuscript for publication. All authors contributed to the article and approved the submitted version.

## Conflict of Interest

The authors declare that the research was conducted in the absence of any commercial or financial relationships that could be construed as a potential conflict of interest.

## Publisher’s Note

All claims expressed in this article are solely those of the authors and do not necessarily represent those of their affiliated organizations, or those of the publisher, the editors and the reviewers. Any product that may be evaluated in this article, or claim that may be made by its manufacturer, is not guaranteed or endorsed by the publisher.
